# Semiconductor-based Multilayer Selective Solar Absorber for Unconcentrated Solar Thermal Energy Conversion

**DOI:** 10.1038/s41598-017-05235-x

**Published:** 2017-07-13

**Authors:** Nathan H. Thomas, Zhen Chen, Shanhui Fan, Austin J. Minnich

**Affiliations:** 1 Division of Engineering and Applied Science, California Institute of Technology, Pasadena, California 91125 United States; 2 Ginzton Laboratory, Department of Electrical Engineering, Stanford University, Stanford, California 94305 United States; 3 Jiangsu Key Laboratory for Design & Manufacture of Micro/Nano Biomedical Instruments, School of Mechanical Engineering, Southeast University, Nanjing, 210096 China

## Abstract

Solar thermal energy conversion has attracted substantial renewed interest due to its applications in industrial heating, air conditioning, and electricity generation. Achieving stagnation temperatures exceeding 200 °C, pertinent to these technologies, with unconcentrated sunlight requires spectrally selective absorbers with exceptionally low emissivity in the thermal wavelength range and high visible absorptivity for the solar spectrum. In this Communication, we report a semiconductor-based multilayer selective absorber that exploits the sharp drop in optical absorption at the bandgap energy to achieve a measured absorptance of 76% at solar wavelengths and a low emittance of approximately 5% at thermal wavelengths. In field tests, we obtain a peak temperature of 225 °C, comparable to that achieved with state-of-the-art selective surfaces. With straightforward optimization to improve solar absorption, our work shows the potential for unconcentrated solar thermal systems to reach stagnation temperatures exceeding 300 °C, thereby eliminating the need for solar concentrators for mid-temperature solar applications such as supplying process heat.

## Introduction

Solar thermal energy conversion is of intense interest due to environmentally sustainable applications in industrial heating, air conditioning, and electricity generation^1–4^. For instance, solar thermal input can be used for industrial process heat instead of furnaces or can replace the compressor in conventional air conditioning units^1, 2^. Solar thermal energy can also be used for desalination of sea water, particularly in remote locations^5^.

A key element of a solar thermal system is a selective surface that simultaneously maximizes solar absorption while minimizing parasitic heat losses due to infrared thermal emission. A non-selective black surface like carbon-black can only reach a maximum temperature of about 130 °C under unconcentrated sunlight. Selective surfaces were originally proposed in the 1950s, and numerous designs have been proposed since, including cermets, metal dielectric structures, and patterned metal surfaces^6–14^. For example, Barshilia *et al*. developed a multilayer stack of Al_x_O_y_/Al/Al_x_O_y_, that is stable up to 400 °C in air and exhibits 96% solar absorptance and 7% thermal emittance^14^. Recently, Cao *et al*. used a calorimetric technique to determine the temperature dependent, hemispherical solar absorptance and thermal emittance of an yttria-stabilized zirconia cermet to be 91% and 13%, respectively at 600 °C^10^.

Another approach for achieving spectral selectivity at moderate temperatures below 500 °C is to use semiconductors with appropriately chosen bandgaps. For photon energies above the bandgap, semiconductors absorb strongly, while for sub-bandgap energies they absorb very little. Because of the near zero absorption below the bandgap, semiconductor based selective surfaces offer the potential for thermal emittance lower than that achieved with surfaces that employ textured metals in the primary absorbing medium. Further, the transition from absorbing to non-absorbing occurs over a very narrow bandwidth compared to that of traditional selective surfaces. Early work by Seraphin and others has demonstrated the potential of using semiconductors for solar thermal purposes^15–18^. However, semiconductors with small bandgaps suitable for absorbing the solar spectrum (0.6 eV to 1.4 eV) have high refractive index and consequently require elaborate antireflection coatings. Standard materials for solar cell antireflection coatings such as SiO_2_, Si_3_N_4_, and TiO_2_ are quite emissive in the mid-infrared, however, and are unsuitable for solar thermal applications. As a result, the performance of semiconductor-based solar thermal absorbers has lagged that of metallic and ceramic counterparts.

In this Communication, we present a semiconductor-based multilayer stack that achieves the high solar absorption and low thermal emission necessary for unconcentrated solar thermal applications. At room temperature, the measured absorption at solar wavelengths is 76%, while at thermal wavelengths the measured absorption is 5%. Variable temperature Fourier Transform Infrared Spectroscopy (FTIR) shows good stability at temperatures up to 300 °C in air, and field tests show a peak operating temperature of 225 °C that is comparable to the performance of state-of-the-art selective surfaces. With straightforward optimization, our surface could achieve temperatures exceeding 300 °C, thereby enabling the use of unconcentrated sunlight for mid-temperature applications such as supplying process heat without need for large geometric concentrators.

## Results

We begin by discussing the constituent materials for a multilayer stack needed to achieve the desired optical behavior. The most important component is the semiconductor that provides the spectrally selective absorption. The semiconductor bandgap energy must correspond to photon wavelength between 1 *μ*m and 2 *μ*m to absorb as much of the solar spectrum as possible. As semiconductors typically have high refractive index, additional materials with lower refractive index must be included in the stack to reduce visible wavelength reflections. For these materials, it is essential that they be transparent at wavelengths longer than the bandgap cutoff wavelength.

Considering these factors, we chose Ge as the semiconductor due to its favorable bandgap energy and CaF_2_ as the dielectric for antireflection. CaF_2_ has low refractive index of about 1.4 and is transparent in the infrared out to 20 *μ*m, making it ideal for antireflection purposes in solar thermal applications^19^. For the primary back reflector, we chose Ag, sandwiched by two thin layers of Cr to improve semiconductor-metal film adhesion.

**Figure 1:**
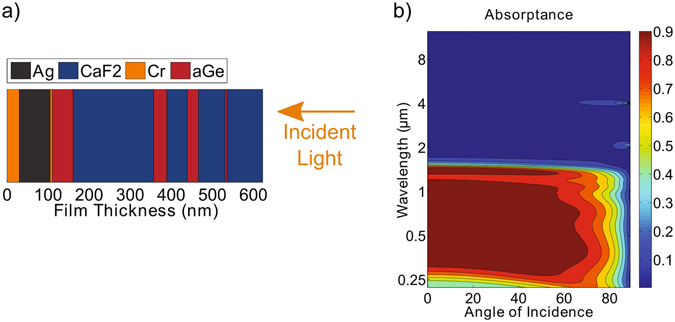
(**a**) Schematic of multilayer stack consisting of Ag, CaF_2_, Cr, and *a*Ge. (**b**) Contour plot of simulated absorptance versus angle and wavelength of incident light. The simulated solar absorptance and thermal emittance is 86.4% and 4.4%, respectively.

Room temperature deposited films of Ge tend to be amorphous, but thin films of amorphous Ge (*a*Ge) have been shown to exhibit temperature stability at moderate temperatures less than 300 °C^20, 21^. Amorphous Ge has different above-bandgap optical properties than those of its crystalline counterpart^22^. Therefore, to model the 1-D stack, we use the bulk refractive index of amorphous Ge but that of crystalline Cr, Ag, and CaF_2_. We optimize the sequence and thickness of thin films of *a*Ge and CaF_2_ for high reflectivity at wavelengths above 1.7 *μ*m and low reflectivity below 1.5 *μ*m using a needles method (see Methods). A schematic of the optimized multilayer absorber is shown in Fig. 1a.

The simulated absorptance of the structure at all incident angles is shown in Fig. 1b. How efficiently a selective surface converts direct sunlight into usable heat is determined by two spectrally averaged quantities. The first is the solar absorptance, *α*_*s*_:

1 $${\alpha }_{s}=\frac{{\int }_{0}^{\infty }\alpha (\lambda ){I}_{AM1.5}(\lambda )d\lambda }{{\int }_{0}^{\infty }{I}_{AM1.5}(\lambda )d\lambda },$$

where *α*(*λ*) is the spectral absorptance and *I*_*AM*1.5_(*λ*) is the AM1.5 spectrum from the sun. The second is the average thermal emittance, *ε*_*t*_:

2 $${\varepsilon }_{t}=\frac{{\int }_{0}^{\pi }{\int }_{0}^{\pi \mathrm{/2}}{\int }_{0}^{\infty }\alpha (\lambda ,\theta ){I}_{BB}(T,\lambda )\,\cos \,\theta \,\sin \,\theta \,d\lambda \,d\theta \,d\phi }{\sigma {T}^{4}},$$

where *I*_*BB*_(*T*, *λ*) is the black body distribution at temperature *T*, and *σ* is the Stefan-Boltzmann constant^23^. Using the simulated absorptance of the structure at all incident angles, shown in Fig. 1b, we calculate the average solar absorptance to be 86.4% and the average thermal emittance to be 4.4%. In particular, the calculated emissivity is lower than those of prior reported works by over 50%, highlighting the potential of semiconductor-based selective absorbers for unconcentrated solar thermal applications for which low emissivity is essential^10, 13^. The solar absorptance can be further increased by introducing additional layers, but for simplicity we consider the stack as designed.

**Figure 2:**
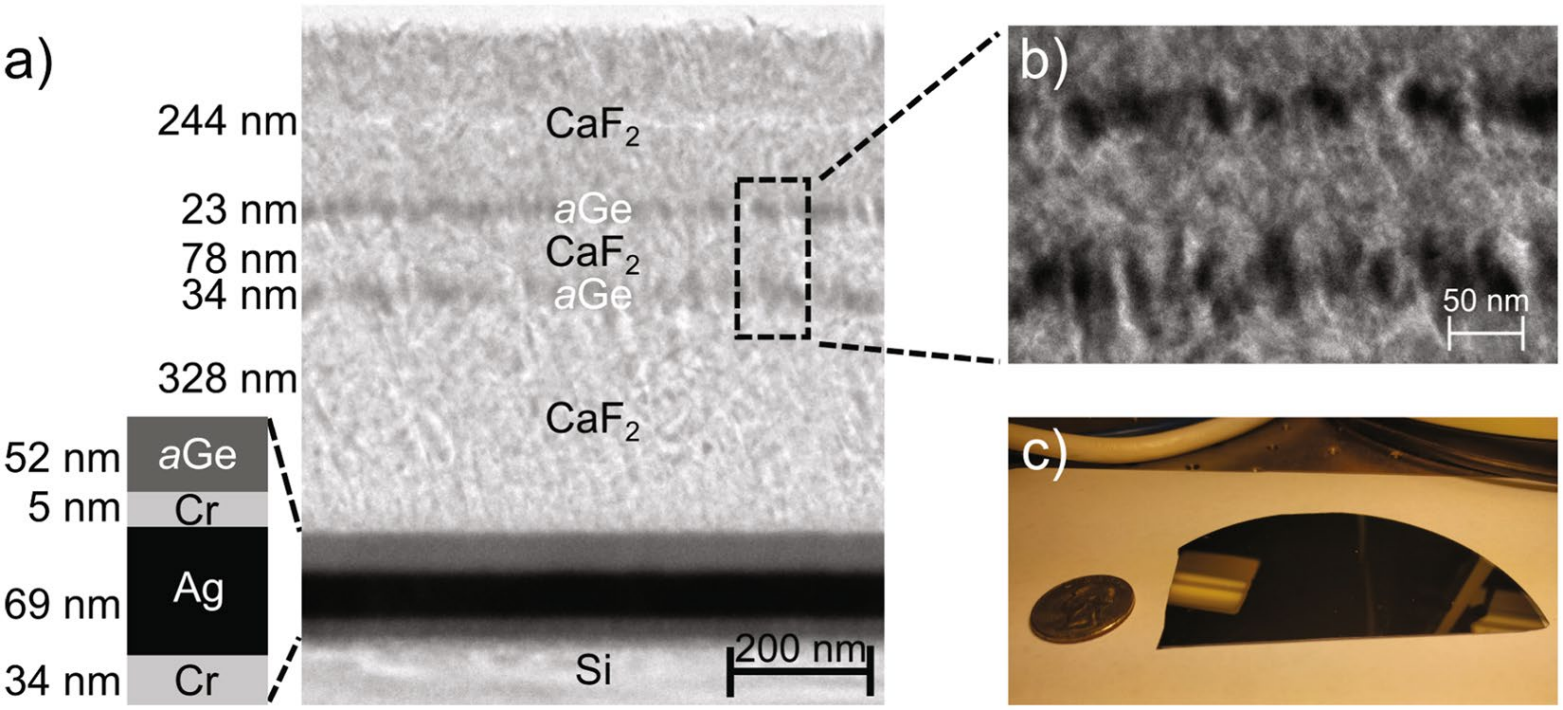
(**a**) Cross section transmission electron micrograph of sample. (**b**) Zoomed in region of CaF_2_ and Ge layers. The gray CaF_2_ layers appear columnar and mix with the black Ge layers. (**c**) Image of sample fragment from 4 inch wafer with U.S. quarter for scale. Sample fabrication is achievable at the wafer scale.

We obtained silicon wafers coated with the multilayer stack from LGA Thin Films, which coated the wafers using electron beam evaporation. We first characterized the structure of the surface using cross-sectional transmission electron microscopy (TEM) in Fig. 2a,b. The first layer of Ge, deposited on top of the Ag layer with the invisible 5 nm layer of Cr in between, is dense. However, subsequent layers of Ge, deposited on CaF_2_, are not dense and mix with the sandwiching layers of CaF_2_. The CaF_2_ appears to form columns that do not provide adequate adhesion sites for the subsequently deposited Ge atoms. As a result, the effective refractive index of the Ge layers that are deposited on the CaF_2_ layers is that of an *a*Ge-CaF_2_ mixture. The designed structure accounted for this mixing by assuming that the refractive index of the initial *a*Ge layer was still that of pure amorphous Ge, but all subsequent layers of Ge had the refractive index of an effective medium whose composition was determined to be 50% Ge and 50% CaF_2_ (see Supporting Information). Further, the deposition rate of CaF_2_ was recalibrated to correct for the discrepancy in thickness between the fabricated surface and the design. A wafer fragment of the final selective absorber is shown in Fig. 2c.

**Figure 3:**
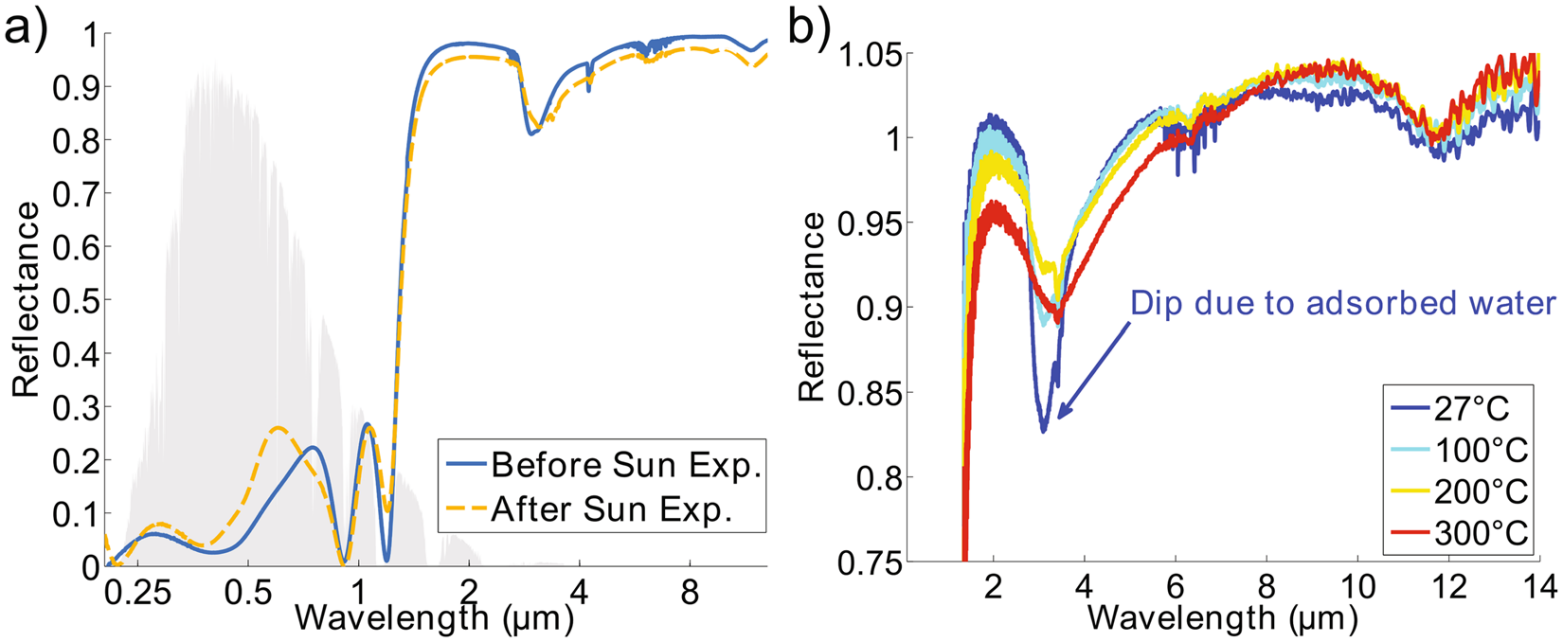
(**a**) Measured reflectance of sample versus wavelength before and after 4 temperature cycles under the sun and 2 cycles under an AM1.5 G solar simulator. The solar spectrum, shown in gray, ranges from 0.28 *μ*m to 2.5 *μ*m, and thermal wavelengths extend beyond 3 *μ*m. Only slight changes in the optical properties are observed after multiple cycles. (**b**) Infrared reflectance versus wavelength at various temperatures. The sharp dip in reflectance at room temperature is primarily due to adsorbed water on the sample surface that evaporates at elevated temperatures. The reflectance exceeds unity due to an absolute uncertainty in the measurement of around 5%.

Next, we measured the room temperature spectral reflectance of the sample using UV-Vis spectroscopy and Fourier-Transform Infrared Spectroscopy (FTIR), as shown in Fig. 3a. Under real-world conditions, incident sunlight is typically close to normally incident, and the spectra in Fig. 3a can be used reliably to determine average solar absorptance. The solar absorptance before exposure to the sun obtained from this measurement is 76%. This value is lower than that of the simulated design due to discrepancies in the actual and targeted thicknesses of the individual layers. Although lower than that of other reported selective surfaces, the solar absorptance can be improved by straightforward optimization of the deposition process.

The infrared reflectance spectra were also measured at temperatures up to 300 °C in dry air, as shown in Fig. 3b. The spectra were taken with a confocal microscope and an HgCdTe (MCT) detector, while the samples were heated on a Linkham FTIR 600 temperature stage with a KBr window. Because of the added KBr window, we found the temperature dependent FTIR spectra depended sensitively on the microscope focus, and the uncertainty of these measurements was 5% absolute. As a result, there are regions in the IR, where the high IR reflectance is shown to exceed unity but by no more than 5%. However, we still use these measurements to determine trends in thermal stability. As the temperature increases, the dip in reflectance at 3 *μ*m becomes less pronounced and is stable from 100 °C to 200 °C. At 300 °C, as indicated by the red curve in Fig. 3b, the reflectance dip at 3 *μ*m begins to decrease again. Interestingly, the temperature cycling seems to initially cause a beneficial increase in IR reflectance. As water is absorptive at 3 *μ*m and these samples were stored in air, a layer of water had likely adsorbed to the surface and evaporated after heating above 100 °C, causing the reflectance to increase^24^. Hence, the room temperature FTIR spectra shown in Fig. 3a, of samples with the adsorbed water layer, indicate a *lower* infrared reflectance than is actually the case. In general, the reflectance drops as temperature increases, as expected, however the total absolute change is about 5% at 300 °C.

**Figure 4:**
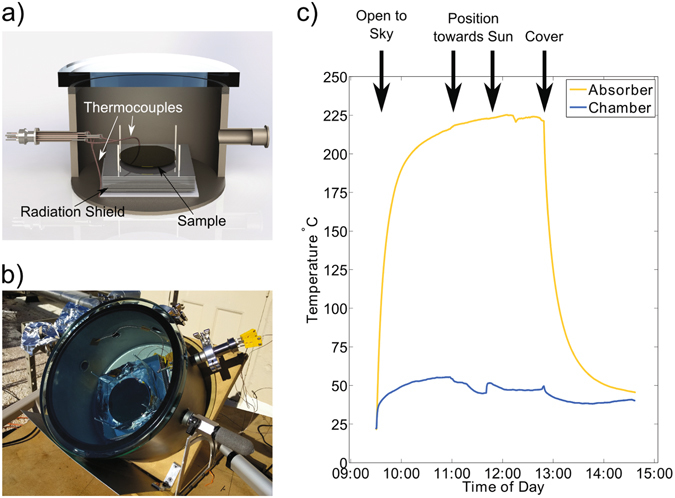
(**a**) Diagram of sample vacuum chamber and radiation shield. (**b**) Photo of vacuum chamber on the roof under solar insolation. (**c**) Sample surface temperature over the course of the day on Dec. 20, 2016 in Pasadena, CA. The temperature peaked at 225 °C.

Determining the hemispherical total thermal emittance requires infrared reflectance measurements over all angles and wavelengths. As these measurements are challenging using readily available equipment, the effectiveness of selective surfaces has been traditionally quantified by a single absorptance or reflectance spectrum, taken at room temperature. A recent work reported a procedure to obtain measurements of average solar absorptance and hemispherical thermal emittance of solar absorbers at operating temperatures^25^. Rather than performing this measurement, we instead determined device performance by measuring the stagnation temperature under solar insolation in field tests in Pasadena, CA. We placed the sample in a 11″ × 11″ vacuum chamber with a glass lid that was in turn placed outside and angled toward the sun, as shown in Fig. 4a,b. We adjusted the angle to ~55° such that the absorber sample was normal to the incoming sunlight. The sample temperature was measured with a type-K thermocouple secured to the surface with either Kapton tape or thermal epoxy. A second thermocouple was also taped to the chamber wall to determine the local temperature of the heat sink. We limited conductive and radiative heat losses by supporting the sample using low thermal conductivity aerogel foam on a radiation shield. The radiation shield was comprised of 10 dual-sided aluminum mirrors separated by low thermal conductivity ceramic washers. The vacuum chamber was pumped continuously to eliminate convective losses and air conductive losses. The chamber was initially pumped to below 1 × 10^−5^ Torr, but as the chamber warmed in the sun, outgassing occurred, leading to a maximum pressure of around 1 × 10^−4^ Torr.

The chamber was positioned to face south and was first exposed to the sun around 9:30am to warm up. Around 11:00am and then again around 12:00 pm, the chamber was positioned to face normal to the sun to maximize absorber temperature. At 1:00 pm after the temperature had plateaued and reached its maximum, the chamber was covered. Measured on Dec. 20, 2016, the absorber temperature peaked at 225 °C.

We also measured the stagnation temperature under insolation from an AM1.5 G solar simulator, under which the peak temperature reached 201.7 °C. Under the same conditions, a commercial state-of-the-art selective absorber reached a peak temperature of 223.1 °C, indicating that our unoptimized sample already achieved performance that is on par with state-of-the-art surfaces.

To determine sample stability, we repeated 6 temperature cycles (4 under the sun and 2 under the AM1.5 G solar simulator) over the course of 20 days, and the surface temperature was measured to be 199 °C or higher in all cases. Shown in Fig. 3a, the solar testing and consequent temperature cycling does cause noticeable changes in reflectance in the UV and in the IR, due to changes in layer morphology at high temperature. However, the *average* solar absorptance, calculated to be 74% after solar insolation, and the average thermal emittance are largely stable, which explains why real-world temperature performance over the 6 independent tests is consistent.

While the performance of our fabricated selective surface compares favorably with that of a state-of-the-art surface, our surface’s stagnation temperature is still considerably below 350 °C, which we predicted from its simulated optical properties. After analyzing our samples, we found this discrepancy could be explained by two reasons. First, the actual layer thicknesses deviated from the target thicknesses, resulting in larger optical reflections than expected. Second, the films of CaF_2_ were porous and did not provide a smooth, homogeneous surface on which the subsequent Ge films could grow. To further improve performance, primarily by improving solar absorptance, optimization of porosity and layer thickness of the CaF_2_ films is necessary. It has been shown that thermally evaporated CaF_2_ films can be grown for optical coatings with high precision^26^. Moreover, ion-assisted deposition of CaF_2_ has been shown to produce layers of smooth, dense films with the refractive index of bulk CaF_2_, necessary for designing absorbers with predictable optical properties^27^. By optimizing these two factors, we predict our surface should achieve a stagnation temperature exceeding 300 °C, with a solar absorptance of around 85%. Such a solar absorptance would be competitive with existing selective surfaces, while the low thermal emittance of our surface would lead to a substantially improved stagnation temperature that would allow unconcentrated sunlight to be used for mid-temperature applications that are presently only achievable using geometric concentrators (see Supporting Information)^11^. In addition, our selective absorber could significantly decrease the area required for rooftop solar thermal systems, thereby facilitating their adoption.

## Conclusion

In summary, we have designed and fabricated a semiconductor based selective solar absorber that exploits the sharp absorption transition of semiconductors at the bandgap energy to achieve high visible absorption yet low infrared emission. In field tests, we obtain peak temperatures consistently exceeding 200 °C, a value comparable to the performance of state-of-the-art surfaces. Straightforward optimization of layer thicknesses and deposition conditions indicate the peak temperature could be increased to 300 °C. Semiconductor-dielectric based selective surfaces can play an important role in expanding the application of unconcentrated solar thermal systems for mid-temperature applications.

## Methods

FTIR measurements were taken on a ThermoScientific FTIR in reflectance mode with a gold reference mirror and a (deuterated triglycine sulfate) DTGS detector from 1.4 *μ*m to 25 *μ*m. For the variable temperature measurement a Linkham FTIR 600 temperature stage with a KBr window and a confocal microscope was used with an MCT detector for wavelengths from 1.4 *μ*m to 15.4 *μ*m. UV-Vis measurements were taken on a Varian Cary5000 UV-Vis Spectrometer with integrating sphere to measure reflectance for 250 nm to 1.8 *μ*m.

The transfer matrix method was used to calculate the reflectance and transmittance. To optimize the structure a needles method was implemented with third party software, OpenFilters^28^.

The films were deposited by electron beam deposition by LGA Thin Films, Inc.

### Data Availability

The datasets generated during and/or analysed during the current study are available from the corresponding author on reasonable request.

## Electronic supplementary material

Supporting Information
