# Supplementary material for: Semiconductor-based Multilayer Selective Solar Absorber for Unconcentrated Solar Thermal Energy Conversion
Source: Sci Rep. 2017 Jul 13;7:5362. doi: 10.1038/s41598-017-05235-x (PMC5509749; doi:10.1038/s41598-017-05235-x)
Supplement: Supplementary file 1 — Supporting Information [file 41598_2017_5235_MOESM1_ESM.pdf]

Supporting Information: Semiconductor-based Multilayer Selective Absorber for Unconcentrated Solar Thermal Energy Conversion

Nathan H. Thomas,[1] Zhen Chen,[2,3] Shanhui Fan,[2] and Austin J. Minnich[1,*]

[1] *Division of Engineering and Applied Science, California Institute of Technology, Pasadena, California 91125,USA*

[2] *Ginzton Laboratory, Department of Electrical Engineering, Stanford University, Stanford, California 94305, USA*

[3] *Jiangsu Key Laboratory for Design & Manufacture of Micro/Nano Biomedical Instruments, School of Mechanical Engineering,Southeast University, Nanjing 210096, China*

(Dated: May 19, 2017)

* aminnich@caltech.edu

JUSTIFICATION FOR EFFECTIVE REFRACTIVE INDEX OF GE LAYERS ON CAF_2

Through the transmission electron micrograph, shown in Figure S1a, we determined the two layers of Ge, labeled as aGe*, were not dense films of pure Ge. Morever, the simulated reflectance, assuming all layers of Ge have the refractive index of bulk amorphous Ge, does not agree with the measured result, as shown in Figure S1b.

As the bottom aGe layer is visibly dense and homogenous in the TEM image, we approximated only the aGe* layers as effective media. The refractive index n_{eff} of those layers was modeled as

$$n_{eff} = n_{aGe}x + n_{CaF_2}(1-x), \tag{S1}$$

where x is the percentage composition of aGe. We found the simulated reflectance spectrum with those layers comprising 50% aGe and 50% CaF_2 is shown to agree well with the measured result. We attribute further small discrepancies between simulation and experiment to surface defects and layer inhomogeneities that scatter incident light. As a result, subsequent optimization of layer thicknesses assumed layers of Ge deposited onto CaF_2 would have an effective refractive index of the 50%-50% mixture.

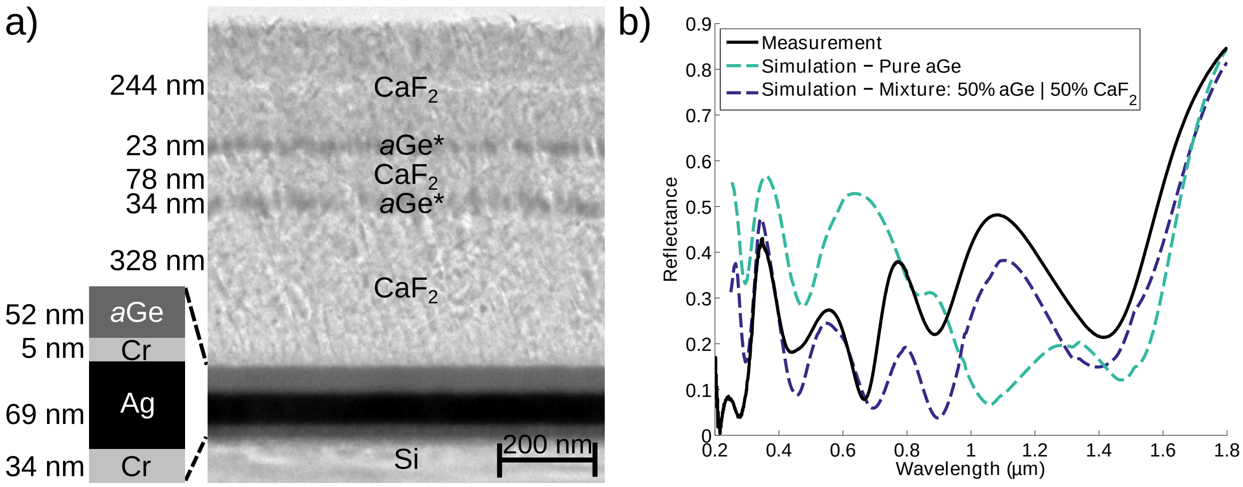

FIG. S1: a) Thickness measurements taken from cross section transmission electron microscopy. b) Measured and simulated reflectance spectra. Using the thicknesses measured in a), we simulate the reflectance spectra for different compositions of aGe-Ca_2 mixtures in the aGe* layers.

ENERGY BALANCE FOR SELECTIVE ABSORBER UNDER SOLAR ILLUMINATION

In this section we describe an energy balance analysis that we use to evaluate the performance of our selective absorber. This model is then used to predict the stagnation temperature of the selective absorber assuming the deposition of fully dense materials and after optimization of layer thicknesses.

We begin by analyzing the radiative heat flux into the selective absorber from the sun and from the surrounding atmosphere,

$$q_{in} = A\int_0^\infty \alpha(\lambda)I_{AM1.5}(\lambda)d\lambda + A\int_0^\pi\int_0^{\pi/2}\int_0^\infty \alpha(\lambda,\theta,\phi)I_{BB}(\lambda,T_{atm})\cos\theta\sin\theta d\lambda d\theta d\phi \tag{S2}$$

where A is the absorber area, $\alpha(\lambda)$ is the spectral absorptance, $I_{AM1.5}(\lambda)$ is the solar spectral irradiance, and $I_{BB}(T_{atm},\lambda)$ is the black body distribution at temperature of the atmosphere T_{atm}. The heat out of the selective surface is both radiative and conductive,

$$q_{out,rad} = A\int_0^\pi\int_0^{\pi/2}\int_0^\infty \alpha(\lambda,\theta,\phi)I_{BB}(\lambda,T_{absorber})\cos\theta\sin\theta d\lambda d\theta d\phi, \tag{S3}$$

$$q_{out,cond} = h_{loss}(T_{absorber} - T_{atm}), \tag{S4}$$

where h_{loss} is the heat flux coefficient between the device and the environment. As the selective absorber is placed on a piece of aerogel foam, we anticipate h_{loss} to be quite low. Further, as the sample is on a radiation shield, the emitted heat out the backside is negligible. The solar absorptance is defined as

$$\alpha_s = \frac{\int_0^\infty \alpha(\lambda)I_{AM1.5}(\lambda)d\lambda}{S}, \tag{S5}$$

where S is the total heat flux from the sun. The thermal emittance weighted by the black body spectrum at temperature T is defined as

$$\epsilon_{t,T} = \frac{\int_0^\pi\int_0^{\pi/2}\int_0^\infty \alpha(\lambda,\theta,\phi)I_{BB}(\lambda,T)\cos\theta\sin\theta d\lambda d\theta d\phi}{\sigma T^4}, \tag{S6}$$

where σ is the Stefan-Boltzmann constant. The net heat flux is therefore

$$q_{net} = C\frac{dT}{dt} = \alpha_s AS - A\left(\epsilon_{t,T_{absorber}}\sigma T^4_{absorber} - \epsilon_{t,T_{atm}}\sigma T^4_{atm}\right) - h_{loss}\left(T_{absorber} - T_{atm}\right), \tag{S7}$$

where C is the thermal capacitance of the absorber. The thermal capacitance is approximated as that of the silicon wafer such that $C = \rho V c_p$, where ρ is the density of silicon,

V is the wafer volume, and c_p is the specific heat of silicon. We adopt the conventional methodology by approximating the second term on the right side of equation S7 as

$$A\left(\epsilon_{t,T_{absorber}}\sigma T_{absorber}^4 - \epsilon_{t,T_{atm}}\sigma T_{atm}^4\right) = A\epsilon_t\sigma\left(T_{absorber}^4 - T_{atm}^4\right), \tag{S8}$$

where ϵ_t is an effective thermal emittance. In general, due to the wavelength selective nature of the absorber, $\epsilon_{t,T_{absorber}}$ does not equal $\epsilon_{t,T_{atm}}$. However as we are operating at moderate temperatures under unconcentrated sunlight, the difference between black body spectra at $T_{absorber}$ is not appreciably different than that at T_{atm} and $\epsilon_{t,T_{absorber}} \sim \epsilon_{t,T_{atm}}$. Therefore, we model the heat flux in and out of the absorber by

$$\frac{dT_{absorber}}{dt} = \frac{A}{\rho V c_p}\left[\alpha_s S - \epsilon_t\sigma\left(T_{absorber}^4 - T_{atm}^4\right) - G\left(T_{absorber} - T_{atm}\right)\right], \tag{S9}$$

where G is the interface conductance between the absorber and the environment.

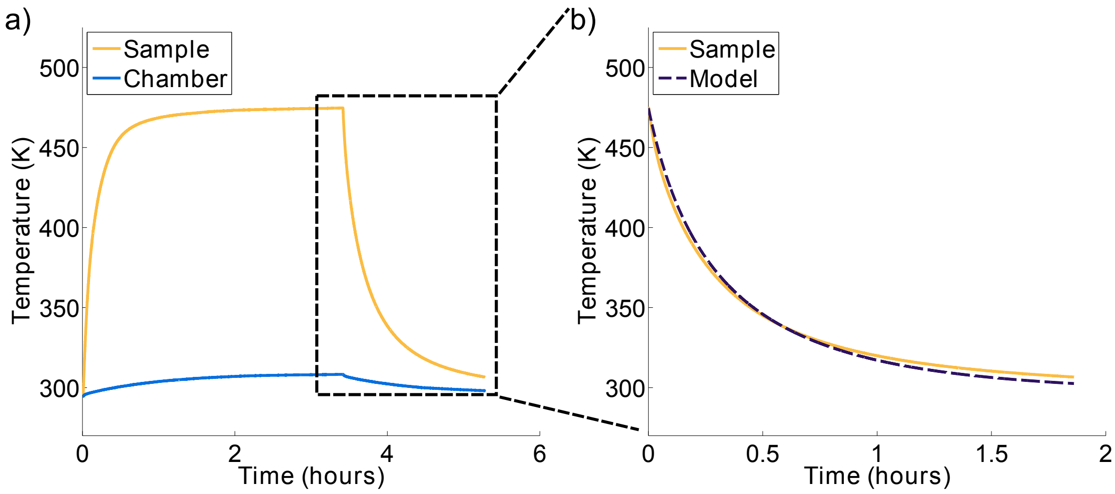

FIG. S2: (a) Temperature data from solar simulator. (b)Measured and modeled temperature decay of sample after the chamber has been covered.

The measured absorber and chamber temperatures over time under illumination from the solar simulator are shown in Figure S2a. We first examine the part of the curve after the sample has been covered and the surface is allowed to thermally decay. In this case, we set $S = 0$ in Equation S9. We then perform a numerical parametric fit for ϵ_t and G for the temperature solution of the differential equation,

$$\frac{dT_{absorber}}{dt} = \frac{A}{\rho V c_p}\left[-\epsilon_t\sigma\left(T_{absorber}^4 - T_{atm}^4\right) - G\left(T_{absorber} - T_{atm}\right)\right], \tag{S10}$$

where T_{atm} is fitted as a spline function to the chamber temperature data. From the parametric fit, we find $\epsilon_t = 0.128$, and $G = 2.809 \times 10^{-6}$ W/m^{2}K. These values are in agreement with our initial assumption that the conductive heat loss would be negligible. The measured and modeled temperature decay are shown in Figure S2b. The marginal discrepancy between the fit and measured data is likely due to the slight temperature dependence of the thermal emittance.

We then use these fitted values to predict the temperature decay for the field test experiment, taken under the sun. The result is shown in Figure S3a. Having validated the model, we calibrate the incident solar flux such that at thermal equilibrium $T_{absorber}$ equals the maximum measured temperature. The simulated temperature rise compared to the measured result is shown in Figure S3b . The simulated temperature rises faster than the measured result because the measurement begins at 9:30AM, when the sun is not high in the sky, and the solar flux is not at its maximum.

Our initial simulations using room temperature optical data show a theoretical solar absorptance of 86% and thermal emittance of 4.4%. With optimization of layer density and thickness, particularly for the CaF_2 layers, we conservatively estimate the solar absorptance could be 85% and thermal emittance to be 7.5%. With these estimates, the predicted temperature curve is shown in Figure S3c with peak temperature of 574 K or 301°C as described in the text. With further optimization of material quality to reduce parasitic infrared absorption, further increase of the stagnation temperature to the theoretical prediction of 350°C could be achieved.

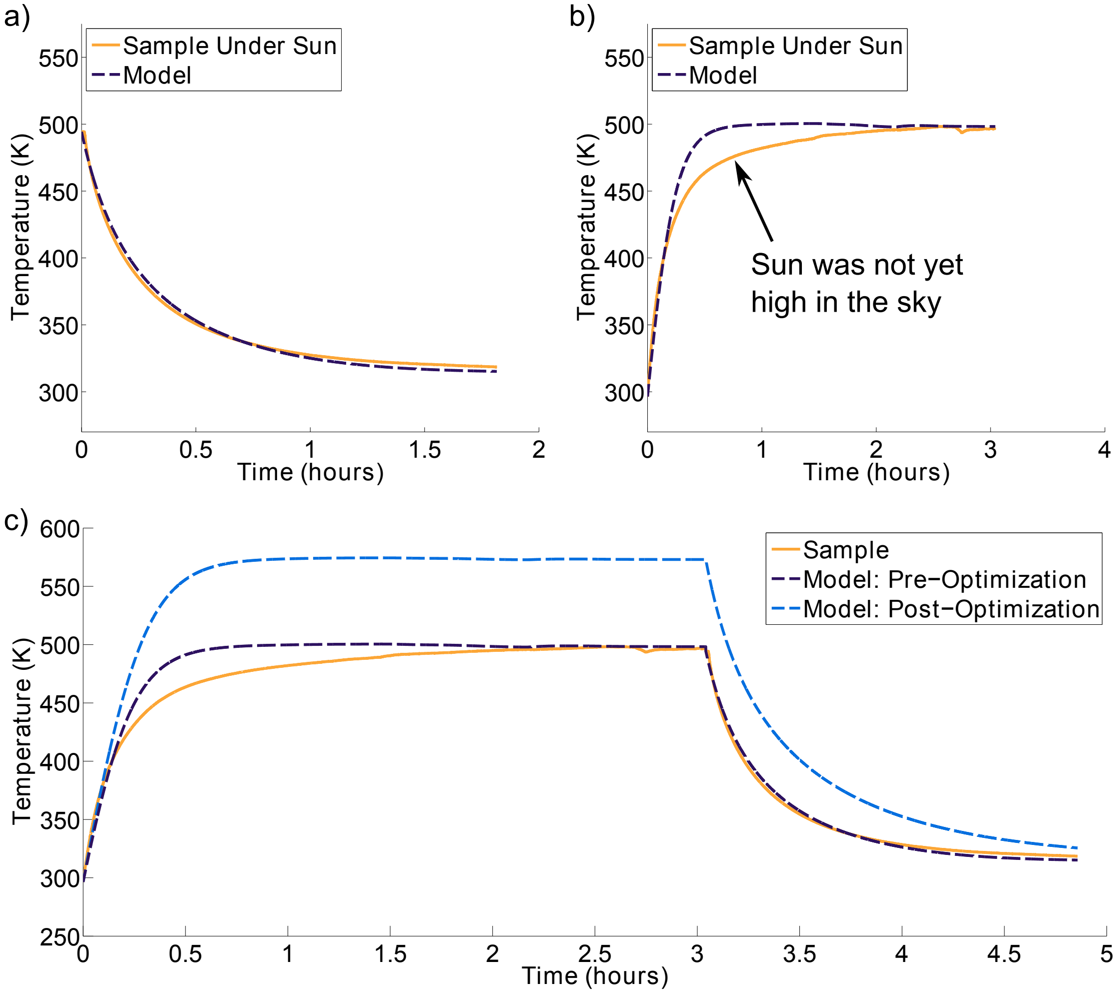

FIG. S3: (a) Measured and predicted temperature decay of sample under the sun, using the fitted values for ϵ_t and G from Figure S2b. (b) Calibrated temperature growth curve. (c) Full modeled temperature rise under the sun. With the optimized values $\alpha_{s,opt} = 0.85$ and $\epsilon_{t,opt} = 0.075$, we predict the peak temperature to reach 574K or 301°C.
